# Documenting research software in engineering science

**DOI:** 10.1038/s41598-022-10376-9

**Published:** 2022-04-21

**Authors:** Sibylle Hermann, Jörg Fehr

**Affiliations:** 1 Institute of Engineering and Computational Mechanics, University of Stuttgart, 70569 Stuttgart, Germany; 2 Cluster of Excellence SimTech, University of Stuttgart, 70569 Stuttgart, Germany

**Keywords:** Engineering, Mechanical engineering

## Abstract

The reuse of research software needs good documentation, however, the documentation in particular is often criticized. Especially in non-IT specific disciplines, the lack of documentation is attributed to the lack of training, the lack of time or missing rewards. This article addresses the hypothesis that scientists do document but do not know exactly what they need to document, why, and for whom. In order to evaluate the actual documentation practice of research software, we examined existing recommendations, and we evaluated their implementation in everyday practice using a concrete example from the engineering sciences and compared the findings with best practice examples. To get a broad overview of what documentation of research software entailed, we defined categories and used them to conduct the research. Our results show that the big picture of what documentation of research software means is missing. Recommendations do not consider the important role of researchers, who write research software, whose documentation takes mainly place in their research articles. Moreover, we show that research software always has a history that influences the documentation.

## Introduction

Documentation of research software^1^ in engineering science is inadequate^2^. Nevertheless, researchers–particularly within the FAIR (Findable, Accessible, Interoperable, Reusable) movement–state that documentation of research software as a major prerequisite for reuse^3^. Although, research data and software play a central role in the Cluster of Excellence “Data-Integrated Simulation Science (SimTech)”^4^, documentation is also lacking here.

But why is research software documented poorly? And what does good documentation actually imply? Previous approaches provide rather explanatory models why documentation is not done; they explain the missing documentation with lack of time^5^ or insufficient training^6^. But is this really the case? Is it even clearly defined what documentation entails? Until now, incentives and rewards are missing for well documented research software. But, the scientific environment is changing, Gil et al.^7^ observe a shift in the scientific environment in different areas: scientific publishing, scientists, public interest and funding. In recent years, these developments are gaining momentum with initiatives like EOSC (European Open Science Cloud)^8^ and NFDI (National Research Data Infrastructure, Germany)^9^. Moreover, research funding agencies demand reusability; the guidelines for good scientific practices, for example, require documentation of research software explicitly^10^. Surprisingly, it remains rather unclear what good documentation of research software involves. We illustrate that there are recommendations on how to document (research) software. But are the recommendations actually applied?

The hypothesis of this paper is, that it is still unclear what good documentation actually involves. The approach intends to examine how documentation takes place in everyday work in a research environment in engineering science within the Cluster of Excellence, SimTech. We also examine if and how given recommendations are implemented. We defined categories to represent different documentation purposes. Based on these categories, we examined three different aspects:

- Given recommendations on how research software should be documented.
- An actual documentation workflow of a specific research software project from engineering science within SimTech.
- Given documentation of two best practice examples within SimTech.

Previous approaches have been concerned with reasons why research software is poorly documented, but not with what good documentation actually entails. Also, it has not been investigated what and how documentation must be implemented in order to be perceived as good. It’s the intention of this approach to show what is missing and give an overview on who has to document for whom what, where, when, and how.

## Methods

We want to investigate how research software is documented in a field where scientists usually don’t have a computer science background. Due to the highly disciplinary nature of research software, we focused on our discipline, Engineering Science. We conducted a multi-case study with a case-based approach^11^. To see how the documentation is implemented, one specific research software was chosen: Neweul-M^2^^12^, a research software that has been developed over years in an institute by engineers without formal software development training. Neweul-M^2^ continues to be actively developed and is often used to address specific research questions. We cross-case synthesis with two other research software’s documentation habits, to compare the gained insights. We selected these best practice examples because they received funding from the DFG (German Research Foundation) sustainable software funding call to improve their documentation^13^. In contrast to Neweul-M^2^, both best practice examples are open source. DuMu^x^^14^ is a research software from engineering sciences, which is also programmed by Research Software Developers with an engineering background. The other example preCICE^15^ is a research software developed from more experienced Research Software Developers working in an informatics institute; their users are mainly engineers. The central rival hypotheses we considered are the lack of time to document and the lack of training of researchers in software engineering^2,6^.

Two main research questions structure the investigation.

### Research questions

1. **What are the recommendations for documenting research software?** Which rules and best practices exist? Do the given recommendations cover the defined categories?
2. **What is the practice of documenting research software?** How is research software documented in the daily life of researchers? Which workflows are implemented? What are the obstacles to document research software?

### Data collection

For collecting data, we choose different sources of evidence:

- **Documentation**: An evaluation of literature was conducted to assess what recommendations are given (RQ1). Furthermore, the three research software documentation were evaluated (RQ2).
- **Participant observation**: Both authors are familiar with Neweul-M^2^, one author from a new Research Software Developer perspective, and the other from years of experience. One author is part of the project from the sustainable software funding call and has thus witnessed the discussions about the possibilities for improvement and shortcomings of the documentation of DuMu^x^ (RQ2).
- **Direct observations**: The concepts, thoughts, and insights were further discussed with the old and later the new Research Software Engineer from Neweul-M^2^ and with DuMu^x^ and preCICE Researcher Software Engineers (RQ2).
- **Interviews**: The two best practice examples were evaluated with semi-structured interviews (RQ1, RQ2).

### Data analysis

Our first idea was to evaluate the research software using given recommendations from the literature. As we soon noticed, the recommendations for research software do not give a complete picture of what documentation should actually contain. Therefore, we switched to an inductive strategy and formed categories that we consider necessary from everyday work with research software, supplemented with categories from literature and internet resources like blogs and wikis. Moreover, we decided to include the best practice examples to answer the research questions. We defined four documentation categories for research software, intending to picture possible documentation forms. Based on the defined categories, we evaluated the recommendations given for, Neweul-M^2^, DuMu^x^, and preCICE. In the following, we introduce the categories, followed by the recommendations and conclude with the analyzed research software examples.

#### Categories

**Domain** Research software can belong to different domains^16^: *private*, *shared* and *open*. Usually, research software is developed in the *private* domain with one main Research Software Developer. The *shared* domain varies from a few users at an institute to many users all over the world, nevertheless the research software is unavailable to the broader public. Published research software in the *open* domain is accessible for everyone. Where *open* can have two different meanings: only the source code is available open source or the software is developed openly. The domains may change over time and require more documentation, as more people need to understand the research software.

**Role** As we noticed, it is essential who documents for whom; we differentiate between three roles: *Research Software Engineer (RSE)*, *Research Software Developer (RSD)* and *user*. One person can have multiple roles, multiple people can share the role and the role of a person can change. As the roles in classical software engineering are conceptualized^17^, we defined the roles from our perspective—which is biased from our education as engineers and work in an interdisciplinary research cluster. When we speak about engineers, we think of the classic engineering fields such as mechanical engineering, civil engineering or chemical engineering. We explicitly neglect software engineers—due to their formal education in software development and maintenance, which is mostly missing in the other fields.

* Research Software Engineers are responsible (i) for the infrastructure and maintenance of the software, (ii) they give the rules of how research software should be written, (iii) but are often not part of the active feature development. The problems of funding, education and missing credit of Research Software Engineers are discussed in the RSE movement^18^.

* Users are (i) research software users who want to do either computer-aided engineering or computer-based experiments without writing code.

* Research Software Developers are (i) the link between *Research Software Engineers* and *users*, (ii) they develop new features, mainly to answer–with the research software–specific new research questions (iii) in engineering without education in software development and are often less experienced than Research Software Engineers. They typically need a specific answer for their research question, for which they need to implement a specific new or missing feature in existing research software. A typical example in Neweul-M2: A RSD implemented the calculation of reaction forces. This new feature can be reused for other research questions from other researchers and, therefore, need to be documented. RSDs are an essential part of the documentation process; they mainly know their developed features but are usually not deeply involved in the maintenance and documentation process.

**Purpose** The purposes describe the content of the documentation: *why*, *what* and *how*. The documentation of the problem should describe *why* the research software or a feature is written–similar to describing the research question, i.e. the RSE Manuscript/Dev docs row from Table 3. The feature’s documentation should describe *what* is needed to be done to solve the research question. *How* the feature is implemented should be documented in a technical documentation, i.e. the Help/Handbook/User Docs row from Table 3.

**Type** The type describes the characteristics of the documentation^19^: *problem*, *product* and *technology*. The three above introduced categories can be expressed in different types of documentation: The documentation of the *problem* can involve how the problem is implemented and why a solution was preferred. The *product* documentation contains the list of all features provided by the software and how they work together. The *technical* documentation should help the RSD and RSE to understand the code, how the research software is engineered and how to build over the existing source code. It should contain different schemas about the used model, the logical, and physical architecture. The types are intended to be umbrella terms for different forms of documentation types. For example, code comments are a form of technical documentation or tutorials as a type of product description.

We assume that each of these categories requires a different type of documentation. In each domain, researchers can document purposes in their role as RSE or RSD for different roles. The combination leads to several types of content, which then appear in a variety of forms. For example, a RSD can describe why they solved what and how in a problem description such as an article. Or a RSE describes how to solve a problem for the user in a product description as a how-to-guide.

**Figure 1:**
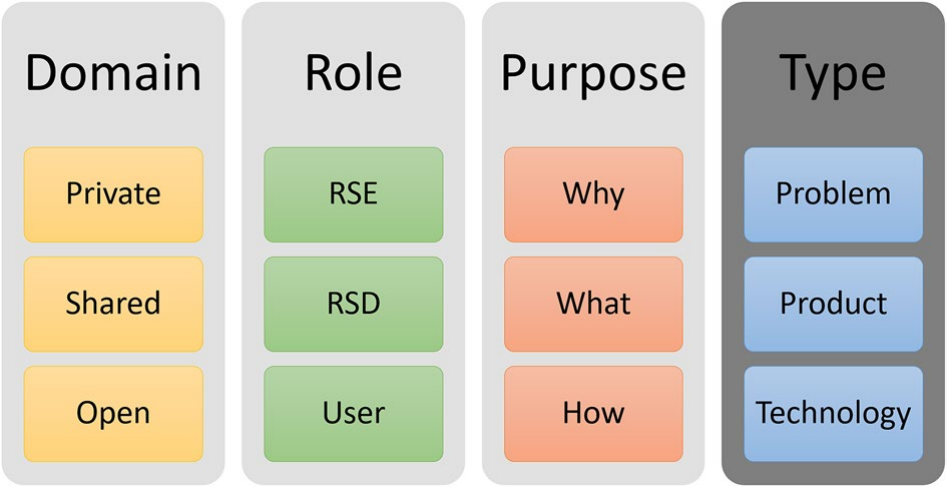
Different aspects of documentation.

#### Recommendations

Aspects of good research software, and its documentation, has also been addressed in various recommendations. In order to find recommendations for documenting (research) software, we conducted a literature review in Web of Science using the terms “research software” and “documentation” or “reusability”. Most articles refer to the whole process of developing research software, and not only to documentation. Often just one small paragraph is dedicated to documentation. The selection of the articles was limited to those that include rules or best practices for documenting research software in at least one paragraph. Ten articles with interdisciplinary and different disciplinary focus were found. As described above, the evaluation of the recommendations did not provide a complete picture of what the documentation in our opinion should contain. Therefore, we also analysed the recommendations according to the categories we defined (Fig. 1).

#### Analysis of research software

**Neweul-M**^2^ Neweul-M^2^ is a software package that allows the dynamic analysis of mechanical systems in calculating multibody systems with symbolical equations^20^. The first version of Neweul was written in FORTRAN with an own symbolic formula manipulator engine in the mid 1970s and was rewritten in 2003 using MATLAB. The new version is called Neweul-M^2^. In Kurz et al.^12^, the history and changes are documented until the year 2010 (for further information see Table 1). Neweul-M^2^ is used from:

- external people (user)
- PhD students (RSD and user)
- students (user)

The source code is developed and administrated by PhD students within the developing institute, they aim for a degree in mechanical engineering and usually don’t have a formal software development education. One experienced RSD is the RSE, a new colleague is briefed as RSE from the previous one.

For the external people and students, a content-obscure (P-code) version of Neweul-M^2^ is provided. One part of the documentation is in an integrated help within MATLAB. The help includes a product description, tutorials and examples and a function reference, automatically generated from the code. For PhD students from the developing institute, the full source code is accessible. The source code is managed via a Git repository hosted at an institutional GitLab instance. Bug fixes and support are the responsibility of the RSE. Another part of the documentation is done in a local wiki with information on how to get started and how to document with coding guidelines, tests and checklists. Decisions and discussions are documented via GitLab. For the RSE, an additional document gives information on how everything is organized. PhD students, who use Neweul-M^2^ for their research, develop new features in Neweul-M^2^ that they need for their research. They document these features mainly in publications.

**DuMu**^x^ The research code for the free and open-source simulator is written in C++ and is based on DUNE (Distributed and Unified Numerics Environment). DuMu^x^ stands for “DUNE for Multi-{Phase, Component, Scale, Physics, ...} flow and transport in porous media^21^. The main intention is to provide a sustainable and consistent framework for implementing and applying of porous media model concepts and constitutive relations (for further information see Table 1). All documentation is linked on the Website. The documentation consists of a collection of documented code examples within the institute’s publicly accessible GitLab instance, a manual in PDF format, code documentation within Doxygen, a reference to the most important publications and a wiki that is still under construction. The software is written by PhD candidates in civil engineering with a predominantly engineering background, who have taught themselves to program.

**preCICE** The research software preCICE^22^ is an open-source coupling library for partitioned multi-physics simulations, including fluid-structure interaction and conjugate heat transfer simulations. The research is about methods how two systems can be coupled (for further information see Table 1). In preCICE the research question is not solved with the software, rather the research results are provided to users of the software. For this reason, there are only RSEs and users. The software is written by PhD candidates with different backgrounds, who are aiming for a degree in computer science. The documentation is bundled in a website. By using GitHub pages, pull requests can be made to all the documentation. The website is divided in quick start, docs, tutorials, community and blog. The section docs start with a user documentation with fundamentals, installation, configuration, tooling and provided adapters. The API is described in the category “couple your code”. Followed by a developer documentation, with a link to the source code documentation in Doxygen, and a description of coding conventions, tooling, workflow and testing. Even a description, how the documentation is build, exists. For the users–in addition to the conventional documentation–a community page gives insights on workshops, other contributors and publications. Furthermore, there is a blog where there is also the possibility to ask questions.

**Table 1:** Information about examined research software projects.

Research software	Neweul-M^2^	DuMu^x^	preCICE
Team size	1 RSE, 3 RSD	2 RSE, 22 RSD	7 RSE
Estimated number of users	40	35	> 100
Year of release	2007	2009	2009
Language	MATLAB	C++	C++

#### Validity analysis

We have deliberately chosen projects in which we can gain a more in-depth insight. These projects are in the engineering field to establish comparability of the training background of RSEs, RSDs and users. The interview partners are based on personal relationships and recommendations, which could lead to a specific research bias due to the small sample size and the personal connection. More extensive studies based on the research hypothesis of this study should be conducted in the future, e.g. at research software conferences. In order to validate our conclusions, we presented and discussed our results and methods with the RSEs and RSDs of the three software projects. We also presented a poster at the internal SimTech conference to receive feedback on our method and conclusions from other researchers. The feedback received confirmed our approach. The poster and other material is published in the case study database^23^. The selection of the case studies initially limits the generalizability of the results. However, the feedback received confirmed that our approach could be transferable to other research software projects. For example, PhD students at the conference confirmed that our approach is similar to their experiences with research software. The generalizability of the findings obtained in this study will be tested in another larger interview study with more extensive surveys in the future.

## Results

We present three main observations from the recommendations and the documentation of three research software examples, based on the categories that are presented in the method section.

### Observation 1: A big picture is missing

A big picture is missing on what documentation of research software should contain and how it should be done. The examined recommendations focus only on specific aspects of research software documentation (see Fig. 1).

### Observation 1.1: Problem and decision are undocumented

The recommendations rarely mention that the why should be documented and seldom take the underlying problem into account (see Table 2). They refer–according to our categories–mainly to the technology of the research software. Their focus is on techniques (like version control systems and programs that generate a documentation out of comments in the code) not on content. Lee^6^ state for example to “use automated documentation tools” and that “the best type of documentation is documentation that writes itself”, but do not explain what have to be the content of the automated documentation. Looking into the practice, all three examples use these automated documentation tools.

In Neweul-M^2^ (see Table 3) the function reference within the help is created with a given template, including: short description, syntax, long description, parameters, examples and references. In DuMu^x^ (see Table 3) the modules are documented with Doxygen^24^. In comparison to Neweul-M^2^ the description involves the underlying concept, mostly explaining the formula behind the code. In preCICE (see Table 3) as well, Doxygen is used with a generic documentation template: the parameters and a one line description is needed and an optional elaborate description. Here, the description do not contain the underlying concept. These tools are intended to document the code not the decisions and problems: RSDs document the problem and decision in research articles or thesis, which are not linked to the documentation; they implement new features to solve a specific task for a thesis; and they describe the problem only in the thesis referring to a specific version of the software. Eventually, the description of the problem and the feature differ from the software solution. Once the feature is included in the main branch, the dependencies are further maintained.

**Table 2:** Purpose mentioned in the recommendations.

Article	Problem	Feature	Implementation
Stodden and Miguez^25^			x
Fehr and Heiland^26^			x
Wilson et al.^27^	x	x	x
Wilson et al.^28^			x
Hastings et al.^29^		x	x
Lee^6^		x	x
Taschuk and Wilson^2^		x	x
Sandve et al.^5^	x	x	x
Karimzadeh and Hoffman^30^			x
Gil et al.^7^		x	x

**Table 3:** Purpose of the different documentations.

Documentation	Neweul-M^2^	DuMu^x^	preCICE
Wiki	F, I	I	
Help/Handbook/User Docs	F, I	F, I	F, I
GitLab/GitHub	P, F, I	F, I	F, I
RSE Manuscript/Dev docs	I		F, I
Publications	P, F	P,F, I	P,F, I
Examples	F	F	F
Code		I	
API			I
Community			F, I
Blog			F, I

P—Problem, F—Feature, I—Implementation.

### Observation 1.2: Shared and private domain are neglected

The recommendations focus on the open domain (see Table 4). Especially, the documentation for the shared domain is rarely mentioned. Neweul-M^2^ is a research software from the shared domain, the different documentation types live in different domains (see Table 5). RSDs document mainly for their successors at the own institute. But the knowledge is not only transported by documentation: students of the institute learn about the software in their lectures and later on from their supervisor and fellow students or colleagues. In the best practice examples, the documentation is openly available.

**Table 4:** Domains mentioned in the recommendations.

Article	Private	Shared	Open
Stodden and Miguez^25^		x	x
Fehr and Heiland^26^			x
Wilson et al.^27^	x	x	
Wilson et al.^28^	x		
Hastings et al.^29^		x	
Lee^6^			x
Taschuk and Wilson^2^	x		x
Sandve et al.^5^	x		x
Karimzadeh and Hoffman^30^			x
Gil et al.^7^			x

**Table 5:** Domains of the different documentations.

Documentation	Neweul-M^2^	DuMu^x^	preCICE
Wiki	S	O	
Help/Handbook/User Doc	S	O	O
GitLab/GitHub	S	O	O
RSE Manuscript/Dev Docs	P		O
Publications	S, O	O	O
Examples	S	O	O
Code		O	
API			O
Community			O
Blog			O

P—Private, S—Shared, O—Open.

### Observation 2: Research software has a history

In engineering science, PhD candidates usually stay round about six years, become experts in a very specific field and then leave. Successors–interested in the same topic–often do not have the chance to talk to them. So the experts omit feedback on their documentation and are ignorant of which questions they have to address in their documentation.

**Figure 2:**
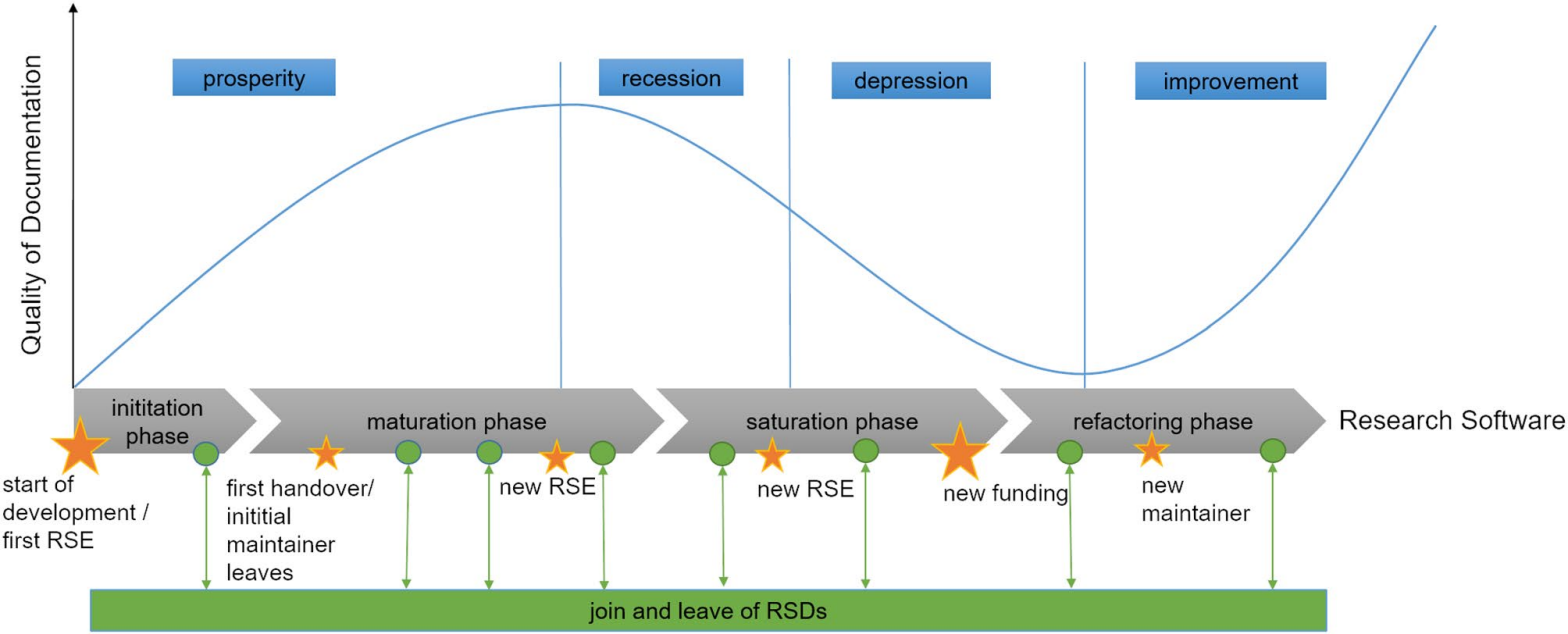
Quality of research software documentation over time. The research software undergoes different phases, from first implementation to continuous application. During these phases, the quality of the documentation varies accordingly. In particular, the change of RSE involves risks, but can also lead to improvements.

Figure 2 describes the observed effect in Neweul-M^2^, showing the quality of the research software documentation over the different phases of research software development. The quality of the documentation behaves similarly to Kondratiev waves^31^: prosperity, recession, depression, and improvement. In the case of Neweul-M^2^ one researcher developed the research software to solve a specific problem in the initiation phase. In the maturation phase, other researchers adopt the research software and more people get involved in the project. At the beginning, the documentation was good (enough) for the people who use the software (prosperity). Eventually, the initial RSD left, and new researchers added new features and modified the code. The quality of the documentation decreased (recession) in the saturation phase because modifications were not documented (depression); until a point was reached where the research software needed a refactoring. A new documentation is needed, usually written with a new tool (improvement). But the old documentation is still used because some aspects are important in there: different documentations exist for different roles in different places with different up-to-dateness. The descriptions of the implemented features in the articles referring to the research software before the refactoring are now more difficult or even impossible to reproduce. A new researcher inherits this history. Comparing this conceptual model with the other research software projects confirms the principle progression; the cycles are more or less pronounced depending on the dynamics of the research software and happen more or less rapidly. New RSEs do not necessarily contribute to the documentation quality. The funding received made it possible to solve many problems in the documentation, which were mainly pointed out by external RSDs and users. They both best practice examples benefit of being open source, receive more feedback from users outside the institute, and spend more effort and money to improve the documentation. So the effect of unconscious knowledge can be minimized.

### Observation 2.1: Unconscious knowledge

In Neweul-M^2^ new users and RSDs are inducted to the software with the help of an more experienced RSD or the RSE. The knowledge of experienced RSDs is often unconscious, that means that they are not aware of their own knowledge and therefore do not explain important steps to the new RSD^32^. The effect shows up for example in the description of the workflow. It is described in the help theoretically but not concretely how and where information is stored and called. Relevant information about what is written in which files are presupposed. Usually, the RSE or experienced RSDs compensates this divergence. For example, where and how storing files is explained in a lecture about Neweul-M^2^, but this information is totally missing in the documentation. In the best practice examples the problem is less pronounced, because users from outside ask questions and draw attention to the problem, and especially in preCICE there is a workflow to update the help, according to the asked questions. Both projects have improved user-friendliness of the documentation through third-party funding. The recommendations do not address this problem.

### Observation 2.2: Missing consistency

In Neweul-M^2^ the documentation lives in different places: An integrated MATLAB help, mainly intended for users; an internal Wiki with more information, mainly intended for RSDs; and an internal document for the RSE with storage locations, workflows and pieces of the history. Not all the documentation is updated with changes in the code. RSDs usually archive their documentation with their thesis in a zip-file. This kind of documentation often refers to an obsolete version, inherited from the history. Moreover, outdated dependencies, which are not documented, invalidate the function or a lot of effort must be spent to fix the dependencies. Looking at the best practice examples: one of the first issues was to have all in one place. Through feedback from external users, the best practice examples are more consistent. Moreover, they spend money and effort to meet these challenges. In DuMu^x^ all the documentation is linked on the homepage, an overview where to find which information is missing. In preCICE the documentation is directly on the homepage, with an overview where to find what. Additionally, there is even meta information about the documentation itself. The recommendations give hints about documentation tools, which can be used–how to structure this information is mentioned in^30^.

### Observation 3: Research software has different purposes

Researchers write research software for different purposes. Therefore, the focus of the documentation can differ as well. The purpose of Neweul-M^2^ is to implement a physical model to evaluate the same effects that occur in or beyond experiments. Engineers use already developed algorithms to solve their research question using research software. Here, in addition to documentation for the users, documentation for the RSDs is also necessary, because the scientists at the institute need to understand the results of their predecessors and want to be able to adapt them to their own needs.

The purpose of DuMu^x^ is similar. However, there is a larger community outside the own institute, which uses the software and develops it accordingly.

The purpose of preCICE is to implement new algorithms and to show that these algorithms work. In preCICE the role of RSDs is not specifically taken into account. Users do couple their software with the help of preCICE but do not contribute to the code with features. So the documentation is mainly intended and improved for scientific users (see Table 6).

**Table 6:** Authors and audience of the different documentations.

Documentation	Neweul-M^2^		DuMu^x^		preCICE	
	Author	Audience	Author	Audience	Author	Audience
Wiki	RSE		RSE	RSD		
Help/Handbook/User Doc	RSE, RSD	User	RSE	RSD, User	RSE	User
GitLab/GitHub	RSE, RSD	RSE, RSD	RSE, RSD	RSD,User	RSE	RSE, User
RSE Manuscript/Dev Docs	RSE	RSE			RSE	RSE
Publications	RSE, RSD	RSE, RSD, (User)	RSE, RSD	RSE, RSD, (User)	RSE	RSE ,(User)
Examples	RSE	RSD, User	RSE	RSD, User	RSE	User
Code			RSE	RSD		
API					RSE	RSE, User
Community					RSE	User
Blog					RSE	User

RSE—Research Software Engineer, RSD—Research Software Developer.

### Observation 3.1: Research Software Developers are neglected

RSDs are often neglected as authors and as audience. As authors of the documentation, the recommendations consider mainly RSEs; the audience are RSEs and users (see Table 7). The documentation requirements for RSDs are unclear. While in practice some requirements are given, the documentation reality often differs. In Neweul-M^2^ the RSE has formalized the documentation of and for the RSDs through a given template. RSDs document directly in the code, which is automatically transferred to the help. The description often remains very short and are sometimes insufficient to be understood by others. No feedback from the successors is given about the quality of the documentation, because the Research Software Developers leave the institute and no longer notice possible problems (see Table 6).

In DuMu^x^ RSDs have as well a guideline how and what they have to document. Experience shows that instead of following the guidelines, RSDs tend to keep the effort as small as possible and do not describe as expected, especially if the requirement is considered unnecessary (see Table 6).

**Table 7:** Authors and audience of documentation mentioned in the recommendations.

Article	Research software engineer		Research software developer		User
	Author	Audience	Author	Audience	Audience
Stodden and Miguez^25^	x	x			x
Fehr and Heiland^26^	x	x			x
Wilson et al.^27^			x	x	
Wilson et al.^28^			x	x	
Hastings et al.^29^	x		x	x	x
Lee^6^	x	x			x
Taschuk and Wilson^2^	x	x			x
Sandve et al.^5^	x	x			x
Karimzadeh and Hoffman^30^	x	x			x
Gil et al.^7^	x	x			x

## Discussion

The results undoubtedly show that research software is documented. We found out that a literature review could not answer RQ1. One hypothesis why we were not able to answer RQ1 from a literature review is Observation 1: The big picture is missing. Who documents what for whom in which domain and for what purpose. The primary hypothesis was that researchers document their software, but that this is not perceived as sufficiently documented. The data collected should provide information about who documents how and why the documentation is not sufficient. The already described rival hypotheses of lack of time and training seemed to be insufficient due to the existing documentation and software knowledge. Our study shows that not necessarily the motivation or missing skills lead to the opinion that software is not documented; rather, research software is not documented as expected.

Often the main problem is that documentation is seen as an event and not a process. Observation 2 shows that the RSEs do not necessarily contribute to documentation quality. Possible reasons for this are different perceptions among the RSEs about what good documentation is, and that old documentation is not discarded. However, funding can improve documentation because they can transform the event character of documentation into a process. The path dependency described in the results as well as the missing consistency and missing framework can be mitigated by setting **uniform standards**. This will always be a balancing act between freedom of research and predefined framework. However, the movement in given structures allows a more efficient work. Also, writing and documenting are not in itself the actual research work, but only the framework in which the research takes place. Researchers have other goals in writing and documenting code than professional software developers. Software developers aim to achieve the objectives defined in the product requirements: Specifying these requirements can be seen as a part of the documentation. Research software, on the other hand, is a means to an end and is not documented in product requirements. Researchers aim to answer research questions with the help of software^33^. Consequently, one part of the documentation happens in research articles, doctoral, master and bachelor theses. Those can be seen as **delayed product requirements**. Nevertheless, this kind of documentation is focused on the research question and not on the research software. Moreover, research papers discuss scientific results based on research software; but the research software behind the results is quickly outdated and developed further. Above all, the **precise implementation of physic** into code in the research software is not specified^34^, but particularly this point is essential for reusing the research software. Besides, articles document research results, not software. Sometimes the material is also **not accessible or difficult to find**. Certainly the lack of time to document is critical, but at a later stage much more time needs to be invested to support the users^30^ and to understand the research software as a RSD. Some papers argue with the lack of training of researchers in software engineering^2,6^. But even in professional software development, documentation is neglected. Ludewig and Lichter^35^ see two reasons for this neglection: Firstly, documentation is not necessarily learned even in software developer training and secondly, although it is said that documentation is important, other aspects usually have priority. Websites like “write the docs”^36^ and The blog “I’d Rather be Writing”^37^ gives advice for technical writers how to document code. Some approaches can certainly be adopted, though not everything can be transferred one-to-one to research software. Initiatives like “Better Scientific Software”^38^ and the “Software Sustainability Institute”^39^ draw attention to the problem and provide assistance. Although these sites are certainly helpful, you need to know them. They give only possible assistance and are not in themselves a standard. Nevertheless, a generally applicable standardization of documenting research software is difficult to find. Existing standards from software engineering^40^ are complex, in parts not relevant for research software and difficult to access. Even if a standardization will be helpful to share results openly, it needs a **clear guideline** to document results for oneself and in a group. Therefore, all three examined research software have some forms of standardized templates, testing strategies and checklists provided by the RSE. However, compliance must also be checked, which in turn costs time. Especially, in engineering science several points add to the described difficulties:

- use of other funding possibilities
- existence of confidentiality reasons
- fear of sabotaging the business model
- modification of existing software, which is unclear how to document

But working together with industry demands good documented results–independent from publishing the software. Moreover, other RSDs need the documentation in order to understand the work from their predecessors. In Neweul-M^2^ RSDs contribute to code and documentation. They have to understand the code, and they have to develop new features, which have to be documented. This part of the documentation can not be written but be controlled by a RSE. RSDs depend on the documentation of their predecessors and the existing structure. This experience happens as well in DuMu^x^. It can also be discussed whether some problems described could be avoided by making the software open source. There are good reasons, such as confidentiality obligations and also often a business model, that make these steps undesirable. Nevertheless, from our point of view, some investigated methods can be transferred to the shared domain.

## Conclusion

All in all, it can be said that it should be clear who documents what and where. Hence, adopting best practices and principles from technical documentation and professional software development can help to improve the documentation of research software. Nevertheless, the study shows that all three case studies struggle with similar problems in the documentation and in part also decided on similar solution strategies, making transferability to other research software projects conceivable. Future research should explore how principles from the best practices examples can be transferred into the shared domain. A possible standardization of content would certainly be helpful here, but this cannot be solved by the individual scientist. The national and international initiatives certainly contribute to improving the situation here. One limitation of the current research is that the findings are not evaluated with more examples. This obstacle can be overcome in evaluating more software documentations. It can be expected that other research software in engineering science has similar problems. Moreover, there is a personal bias when trying to solve the problem with a given documentation. The experience showed that it was totally clear for the Research Software Engineer of the help where they can find the information and how the documentation is structured. But for inexperienced users, it is not obvious, they have to ask the Research Software Engineer. The effort to write documentation should be taken into account. Will the benefit exceed the effort that must be used for documentation? This can be an area for future research. As long as people are there who can help, it is just inefficient but not impossible to solve the given task without a sufficient documentation. But in the current discussion about FAIR, research software documentation plays an important role. The pay-off for Research Software Developers is may be marginal at the moment, but the importance is increasing. Good documentation pays off in the long run.

We discovered that researchers are often not aware for whom and why they document. A big picture what documentation for research software means is missing. The new approach in this paper is to define for what purpose and what appearance the documentation is intended and who has to document what for whom depending on the domain. The paper shows that even in recommendations, the objective of the documentation of research software is unclear. Until now the focus lies on Research Software Engineers and user, the researcher who develops features to an existing research software is here brought into focus. While essentially only the open domain has been considered so far, a substantial part of research software does not take place publicly in the first place; here, too, documentation is needed in order to ensure sustainable research.
